# Current Hospital Topics

**Published:** 1907-03-23

**Authors:** 


### HOSPITAL ADMINISTRATION.

### CONSTRUCTION AND ECONOMICS.

### CURRENT HOSPITAL TOPICS.

#### Mr. H. Howgrave Graham.

After twenty-five years of zealous service, in which he has shown great devotion, Mr. H. Howgrave Graham has resigned the secretaryship of the Hospital for Epilepsy and Paralysis. The vacancy was advertised in The Hospital last week, and we are glad to note that the Committee have determined to select some one to succeed Mr. Graham, who has had training and experience in the work. Mr. Graham did yeoman service when this hospital was removed from Regent's Park to Maida Vale, and the Committee have properly shown Mr. Graham extreme kindness and warm appreciation of the great services he has undoubtedly rendered to this institution. Mr. Graham decided to resign, as he wished to diminish the amount of his work, and to free himself from the worries of hospital life, inseparable from its nature, however pleasant may have been the secretary's relations, as in Mr. Graham's case, with the staff and others connected with the institution. We wish Mr. Graham the best of health, a long life and continued energy to carry on his other appointment, that of secretary and registrar to the Chartered Institute of Patent Agents, to which he will devote much more time for the future. We have known Mr. Howgrave Graham for more years than we care to remember, and have always found him anxious to do his work thoroughly, to master fully every branch of it, to enforce economy, and to attract revenue from new sources. In the latter art he has displayed great ingenuity in thinking out plans which have yielded no small advantage to the various institutions for whose benefit they were conceived.

#### The Bournemouth Hospitals.

We are glad to see that at the Annual Meeting of the Royal Victoria Hospital, Bournemouth, the question of amalgamating that institution with the Royal Boscombe Hospital was under consideration. We expressed our opinion in favour of such a step, and the reasons upon which it is based, on a former occasion. Lord Malmesbury was justified, on the facts, in declaring that Bournemouth is too much tapped, owing to the flooding of its residents with a variety and number of charity appeals of all sorts and kinds, many of which have little, or nothing, to do with this popular watering place. We are glad to note that, the *Bournemouth Guardian* supports the amalgamation of the two local hospitals, and that it urges the managers of these institutions to "arrive at a happy working basis," whereby the town would be greatly benefited and economy in administration promoted. The occasion offers an opening to a man of affairs, with local influence, who will devote time and tact on public grounds, to the amalgamation of these hospitals, in the interests of the public, the profession and the suffering poor. In any case, we hope that the inhabitants of Bournemouth will refuse to waste their funds, by providing money to erect a branch hospital at the north-east end of the town. By such a course every intelligent citizen, with means, has an opportunity to throw his weight into the scale of promoting the interests of the whole community, as opposed to the interests or predilections of a few residents, however worthy and enthusiastic the latter may be. If Bournemouth residents keep a tight hand on their purse strings, amalgamation and great economy will be brought about without doubt, or prolonged delay.

#### Annual Donations v. Sustentation Funds.

The raising of the revenue of a voluntary hospital is a business, and the greater the experience possessed by the secretary, the more flourishing the revenue becomes. This is one reason, why no committee which is alive and awake,—except where sometimes a local candidate of influence without special experience may prove a better revenue raiser than a stranger, however experienced,—will, nowadays, be content to select for the post of Hospital Secretary someone without training for the post, until they have exhausted every effort to secure such a candidate. The idiosyncrasies of subscribers and donors must be recognised, or the revenue suffers. There are many people who, while refusing to give annual subscriptions, in fact do contribute every year to particular hospitals by sending donations. A capable secretary keeps a book of annual donors, and is careful to write to them each year about the time the last contribution was received. In this way in the course of years a large and increased sustentation fund may be built up. At Bradford, as at the London Hospital, the Royal Infirmary has raised a sustentation fund of £15,000, extending for a period of five years. That period expires in 1908, and it has been decided to make a special effort to obtain a renewal of these donations for a further period of five years. We incline to the opinion, formed by experience, that sustentation funds are a mistake. They have a tendency to make a hospital unpopular because of the strained efforts which are required, periodically, to keep them going. There is no real attraction, in a financial sense, in announcing that a fund of say, £50,000 or £100,000 has been raised by five years' sustentation-funds. Rather does the hospital suffer, by creating a belief that it is in funds, whereas it has in fact only organised limited contributions of £10,000 to £20,000 a year from certain donors. Good hospital finance merely entails an organisation of a variety of means, which, collectively, will yield during the year about the sum required to meet the current expenditure. Any voluntary hospital, in the present day, which restricts its relief to deserving cases, and has a capable administrator for its secretary, should be able to get ample funds and to extend its popularity with the public every year. Sustentation funds may produce opposite results. We should therefore, advise the Mayor of Bradford to consider the question, in these varying aspects, before committing the town to another sustentation fund.

